# Supplementary material for: High-dimensional multiomics reveals perturbations to IL-6/IL-6R axis and RUNX3 in CD4+ T cells during third trimester pregnancy
Source: bioRxiv. 2026 Mar 30:2026.03.26.711478. Preprint. [Version 1] doi: 10.64898/2026.03.26.711478 (PMC13060162; doi:10.64898/2026.03.26.711478)
Supplement: Supplement 2 [file NIHPP2026.03.26.711478v1-supplement-2.pdf]

bioRxiv preprint doi: https://doi.org/10.64898/2026.03.26.711478; this version posted March 30, 2026. The copyright holder for this preprint (which was not certified by peer review) is the author/funder, who has granted bioRxiv a license to display the preprint in perpetuity. It is made available under aCC-BY 4.0 International license.

Supplementary Figure 1. Gating strategies for LEGENDScreen and scRNAseq. (A) Gating strategy from pre-processed flow cytometry data as previously described[25] to analyse T_{Naive}, T_{SCM}, T_{CM}, T_{EM}, T_{EMRA}, and T_{reg} $CD4^+$ T cell subsets in LEGENDScreen. (B) Gating strategy to sort on T cells for single-cell RNA sequencing, of which $CD4^+$ T cells were determined bioinformatically from RNA sequencing.

Supplementary Figure 2. Gating strategy for validation flow cytometry. (A-B) Gating strategy to analyse selected differentially expressed markers on peripheral blood $CD4^+$ T cells (A) and decidual $CD4^+$ T cells (B). Gates were set on the $CD45^+$ population to define thresholds before applying them to $CD4^+$ T cells.

(which was not certified by peer review) is the author/funder, who has granted bioRxiv a license to display the preprint in perpetuity. It is m
available under aCC-BY 4.0 International license.

A

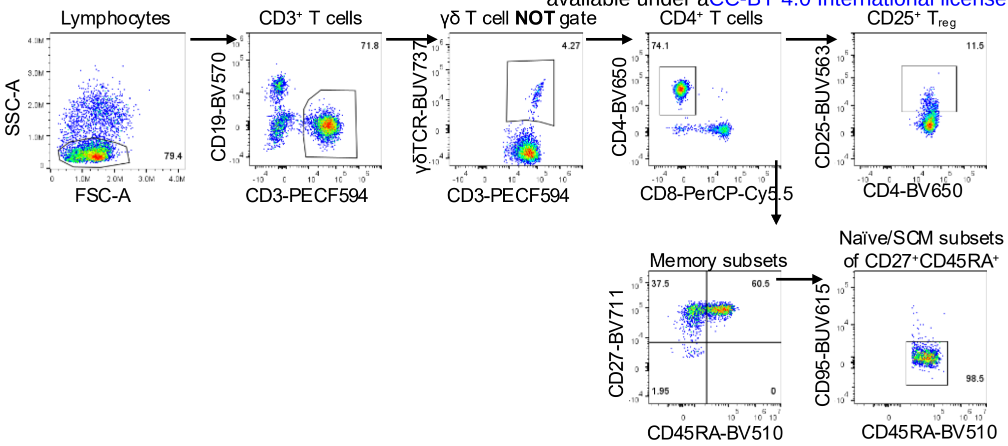

B

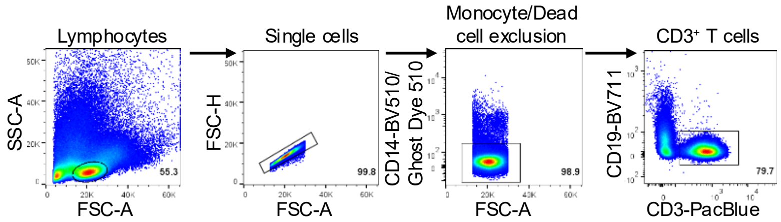

Habel et al Supplementary Figure 1

(which was not certified by peer review) is the author/funder, who has granted bioRxiv a license to display the preprint in perpetuity. It is m
available under aCC-BY 4.0 International license.

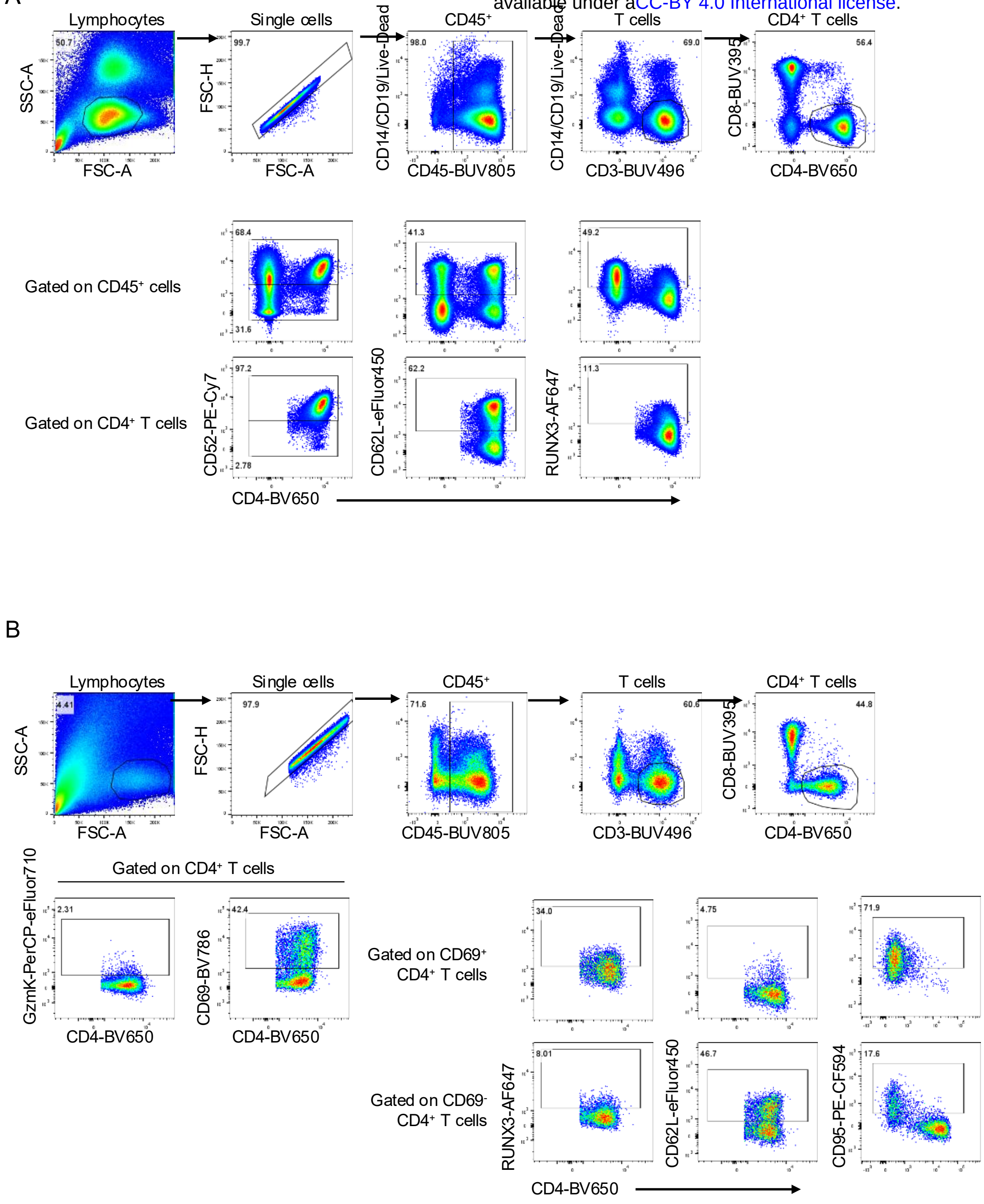

Habel et al Supplementary Figure 2
